# Supplementary material for: HERV-Derived Ervpb1 Is Conserved in Simiiformes, Exhibiting Expression in Hematopoietic Cell Lineages Including Macrophages
Source: Int J Mol Sci. 2021 Apr 26;22(9):4504. doi: 10.3390/ijms22094504 (PMC8123466; doi:10.3390/ijms22094504)
Supplement: Supplementary file 1 [file ijms-22-04504-s001.zip › Supplementary information1_0421AM.pdf]

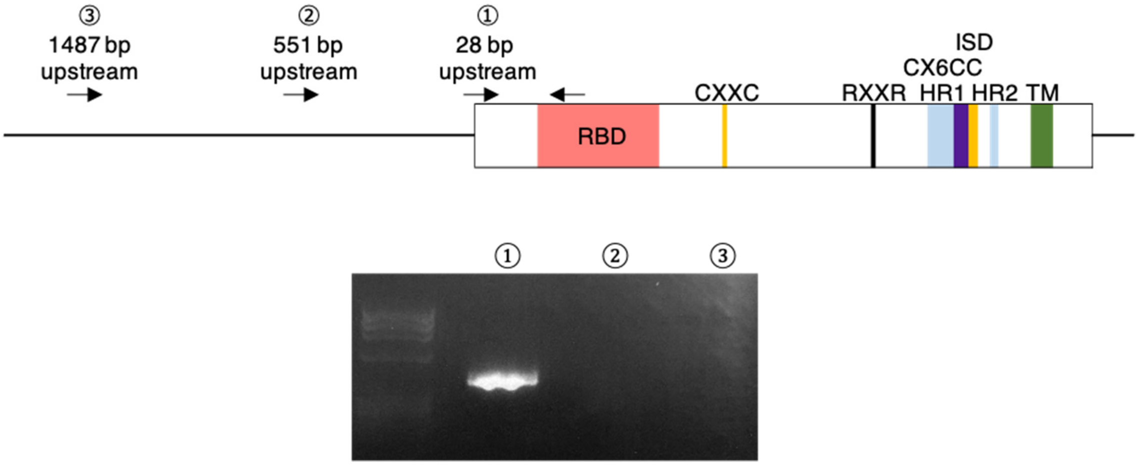

Figure S1. Confirmation of ERVPb1 upstream sequence by RT-PCR. The upper figure shows the positional relationship between the ERVPb1 ORF and the three primer sets. Primer sets 1-3 contain 28, 551 and 1487bp upstream of ERVPb1 ORF, respectively. The lower figure indicates the results of RT-PCR. The RT-PCR primer used were UTR-F1: AAACTGCAGTCTGAACTGCCGAGAA, UTR-F2: AGAAAACACTGACTCAGACAACAGG, UTR-F3: CCAGTTATATACCTGGGTCGAATTAATGTCCA and ORF-R: GGGGACCCAATCTTGACGTTGAGTG.
