# Supplementary material for: HERV-Derived Ervpb1 Is Conserved in Simiiformes, Exhibiting Expression in Hematopoietic Cell Lineages Including Macrophages
Source: Int J Mol Sci. 2021 Apr 26;22(9):4504. doi: 10.3390/ijms22094504 (PMC8123466; doi:10.3390/ijms22094504)
Supplement: Supplementary file 1 [file ijms-22-04504-s001.zip › Supplementary information2.pdf]

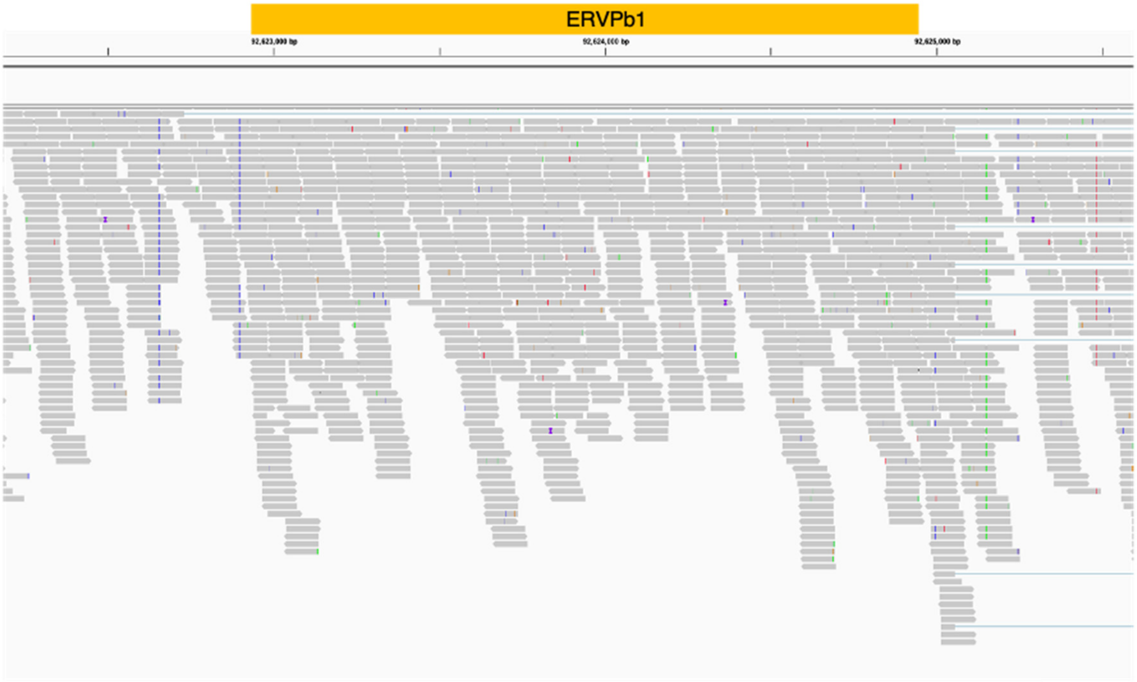

Figure S2. IGV browser views of ERVPb1 locus. Human primary monocytes using short-read RNA-seq data registered in the European Gnome-phenome Archive (EGA: accession code EGA: EGAS00001001895) from healthy 200 individuals (each 100 Africans and Europeans). Gray box and color bars indicate each read sequence and mutations to adenine (green), guanine (brown), cytosine (blue) and thymine (red).
